# Infection-Mediated Clinical Biomarkers for a COVID-19 Electrical Biosensing Platform

**DOI:** 10.3390/s21113829

**Published:** 2021-06-01

**Authors:** Reena Sri Selvarajan, Subash C. B. Gopinath, Noraziah Mohamad Zin, Azrul Azlan Hamzah

**Affiliations:** 1 Institute of Microengineering and Nanoelectronics (IMEN), National University of Malaysia (UKM), Bangi 43600, Malaysia; dr_reena@ukm.edu.my; 2 Institute of Nano Electronic Engineering (INEE), University Malaysia Perlis (UniMAP), Kangar 01000, Malaysia; subash@unimap.edu.my; 3 Center for Diagnostic, Therapeutic and Investigative Studies, Faculty of Health Sciences, National University of Malaysia (UKM), Kuala Lumpur 50300, Malaysia; noraziah.zin@ukm.edu.my

**Keywords:** cytokine storm, pro-inflammatory cytokines, acute phase proteins, adjunct diagnostic biomarkers, prognostic indicators, electrochemical sensors, electronic biosensors

## Abstract

The race towards the development of user-friendly, portable, fast-detection, and low-cost devices for healthcare systems has become the focus of effective screening efforts since the pandemic attack in December 2019, which is known as the coronavirus disease 2019 (COVID-19) pandemic. Currently existing techniques such as RT-PCR, antigen–antibody-based detection, and CT scans are prompt solutions for diagnosing infected patients. However, the limitations of currently available indicators have enticed researchers to search for adjunct or additional solutions for COVID-19 diagnosis. Meanwhile, identifying biomarkers or indicators is necessary for understanding the severity of the disease and aids in developing efficient drugs and vaccines. Therefore, clinical studies on infected patients revealed that infection-mediated clinical biomarkers, especially pro-inflammatory cytokines and acute phase proteins, are highly associated with COVID-19. These biomarkers are undermined or overlooked in the context of diagnosis and prognosis evaluation of infected patients. Hence, this review discusses the potential implementation of these biomarkers for COVID-19 electrical biosensing platforms. The secretion range for each biomarker is reviewed based on clinical studies. Currently available electrical biosensors comprising electrochemical and electronic biosensors associated with these biomarkers are discussed, and insights into the use of infection-mediated clinical biomarkers as prognostic and adjunct diagnostic indicators in developing an electrical-based COVID-19 biosensor are provided.

## 1. Introduction

**Figure 1:**
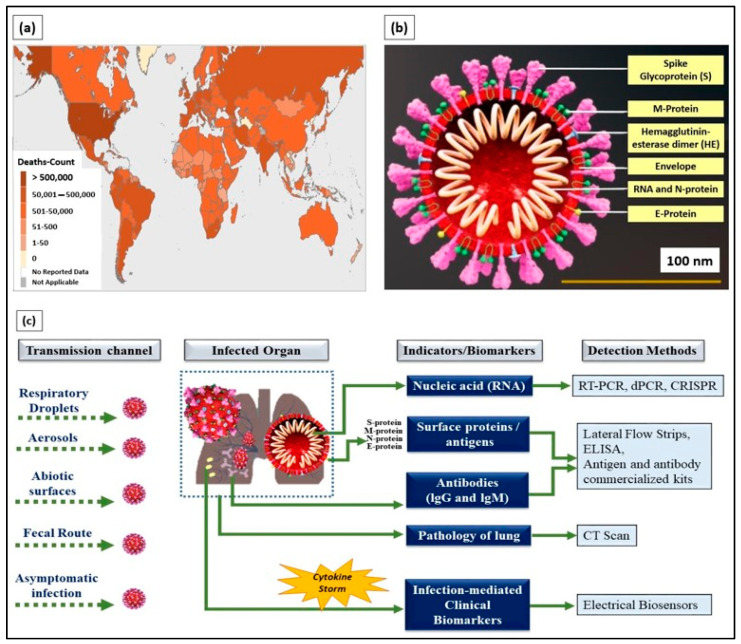
(**a**) World Health Organization (WHO) coronavirus disease 2019 (COVID-19) dashboard (choropleth map) indicating death count globally as of the 6th of May 2021 (4.43 pm CEST). (**b**) Cross-section of SARS-CoV-2 virus structure (picture credits: Scientific Animations/Wikimedia Commons). (**c**) Current existing indicators and biomarkers with transmission channels and detection methods are illustrated.

The coronavirus disease 2019 (COVID-19), an infectious disease that caused a global pandemic, originated from the causative virus “SARS-CoV-2”, also known as the coronavirus. With the initial outbreak in Wuhan, China, in November 2019, the World Health Organization (WHO) declared 155,665,214 confirmed cases and 3,250,648 deaths globally as of the 6th of May 2021 (4.43 pm CEST). The detailed death counts are shown in the choropleth map of Figure 1a. This single-stranded and positive ribonucleic acid (RNA) viral strain originated from the subfamily Orthocoronavirinae [1]. Its genome has a size range of 26–32 kilobases, which has ~80%, ~50%, and ~96% similarity to the genomes of SARS-CoV, the Middle East respiratory syndrome virus (MERS-CoV), and bat coronavirus RaTG13, respectively [2].

Typically, SARS-CoV-2 viral genomic RNA is enveloped by membrane proteins and has embedded spike (S), envelope (E), nucleocapsid (N), and hemagglutinin esterase (HE) [3] proteins (Figure 1b). Upon entering the human body through respiratory droplets, aerosols, abiotic surfaces, fecal routes, and asymptomatic transmission [4], the SARS-CoV-2 virus binds to the alveolar epithelium in the lungs with the help of the angiotensin-converting enzyme-2 (ACE2) host receptor [5]. This, in turn, activates the immune system [6] and induces a “cytokine storm” as more immune cells attack the virus.

Infected patients exhibit a wide range of symptoms, including cough, sneeze, and fever, which are commonly observed in most patients. Moreover, a definite number of asymptomatic and pre-symptomatic infected patients have contributed to the bloom of daily cases around the globe. The continuous alteration of the genetic material on SARS-CoV-2 through mutation exacerbates the current condition. The D614G spike-mutated SARS-CoV-2 discovered in July 2020 is 10 times as infectious as its predecessor (D614) [7]. The emergence of another mutated COVID strain (B.1.1.7) from the United Kingdom (UK) revealed the capability of SARS-CoV-2 to continuously modify its genetic information [8].

Owing to its deleterious nature, it has enticed researchers, scientists, physicians, virologists, and multidisciplinary experts around the world to collaborate and play their roles in handling the current situation. Currently, the distribution of COVID-19 vaccines from Pfizer, Moderna, SINOVAC, Sputnik V, and the University of Oxford has restored hope worldwide. Amid this release, researchers are working continuously on providing better solutions or finding an endpoint to restore the world into its pre-pandemic conventional state.

Currently, detection methods based on biological indicators, such as ribonucleic acid (RNA), viral proteins (e.g., S, M, N, and E), the response of the body’s immune system (antibody- and antigen-based responses), and clinical imaging (e.g., chest X-rays and CT scans), have been used in diagnosing COVID-19 [9] (Figure 1c). These methods are the gold standard and provide prompt solutions for diagnosing infected patients. However, laborious RNA extraction protocols, lengthy assays, prolonged time consumption [10], the need for centralized laboratories and trained laboratory technicians [11], and alterations, especially mutations in viral RNA sequences, are the key factors limiting the use of nucleic acids as indicators for COVID-19 diagnosis.

Meanwhile, antigen-based detection is only applicable for diagnosis in symptomatic patients with high viral loads compared with patients at the initial stage of infection. Often, it leads to an increasing rate of false negatives in low-viral-load patients, causes super-spreading, and contaminates the environment that had been previously been kept virus free [12]. Access to CT scan imaging facilities is limited to large centralized hospitals and is limited in terms of specificity toward SARS-CoV-2 infection, as COVID-19 cannot be distinguished from other infections through CT scan [4]. Therefore, adjunct indicators for detecting pre-symptomatic and symptomatic patients with false negatives in epidemic surroundings should be explored.

Moreover, suitable prognostic indicators are necessary for evaluating the severity of an infection and are vital to the management of an outbreak. In particular, personnel from hospitals and clinical research centers need a solid indicator to retrieve data on the prognosis of COVID-19. Prognostic indicators are essential to the understanding of the nature of SARS-CoV-2 infection, the formulation of strategies for prompt treatment, and the development of effective drugs and vaccines.

Therefore, infection-mediated clinical biomarkers released by the body’s immune system can be used as prognostic indicators of COVID-19. The elevated concentrations of these biomarkers in infected individuals can be monitored and used in evaluating the severity of the illness. Elevated concentrations of infection-mediated clinical biomarkers provide a better understanding of the nature of the infection and aid in developing efficient vaccines that can support clinical research on handling the current pandemic. In addition, certain biomarkers, especially interleukin-6 (IL-6), serum ferritin, and serum amyloid A (SAA) released during an immune response or “cytokine storm”, can be used as adjunct indicators for COVID-19 diagnosis, particularly for symptomatic patients, but show false negative results in conventional and “gold-standard” detection methods. Pre-symptomatic and symptomatic patients from epidemic surroundings can be diagnosed by monitoring the secretion range of these biomarkers in their bodies.

Therefore, the most promising tools for detecting these infection-mediated clinical biomarkers are biosensors. Biosensors are analytical devices composed of a biorecognition element and a physical transducer that converts a recognition phenomenon into a measurable signal [13]. Typically, biosensing platforms offer ultra-sensitivity, high selectivity, cost effectiveness, easy fabrication, and a quick turn-around time for the detection of pathogens and biomolecules [14,15]. In particular, electrical-based biosensors comprising electrochemical and electronic biosensors allow for direct translation of the conjugation of targets with probes into electrical signals. Hence, a fast response time and easy operation are achieved, paving the way for the application of the biosensors in point-of-care (POC) tests.

The first biosensor for the accurate detection of SARS-CoV-2 was reported by Qiu et al. [16] in April 2020. The researchers implemented dual-functional plasmonic biosensors by combining the plasmonic photothermal effect and localized surface plasmon resonance for precise SARS-CoV-2 detection, with a limit of detection (LOD) value of 0.22 pM. Seo et al. [17] reported a graphene-based field-effect transistor (GFET) biosensor for the rapid detection of SARS-CoV-2. The GFET exhibited an LOD value of as low as 1.6 × 101 pfu/mL and 2.42 × 102 copies/mL in the culture medium and clinical samples, respectively.

The biosensors mentioned above mainly detect nucleic acids and surface proteins as biomarkers for COVID-19 diagnosis. However, repurposing the roles of infection-mediated clinical biomarkers released due to “cytokine storm” demonstrated the potential of the biomarkers as prognostic and adjunct diagnostic indicators for COVID-19 infection. Therefore, detecting and monitoring these infection-mediated clinical biomarkers facilitates severity analysis of the illness and provides additional options for diagnosing infected patients.

Thus, this review reports and highlights potentially effective infection-mediated clinical biomarkers as adjunct diagnostic and prognostic indicators of COVID-19. The claim for each infection-mediated clinical biomarker is supported with clinical data obtained from studies of the blood chemistry of COVID-19 patients. Recently reported works on electrical biosensors associated with these COVID-19 biomarkers are reviewed, and insights into infection-mediated clinical biomarkers for building electrical devices for future POC applications are provided at the end of this review.

## 2. Pathophysiology of SARS-CoV-2 Infection and Clinical Types of Patients

**Figure 2:**
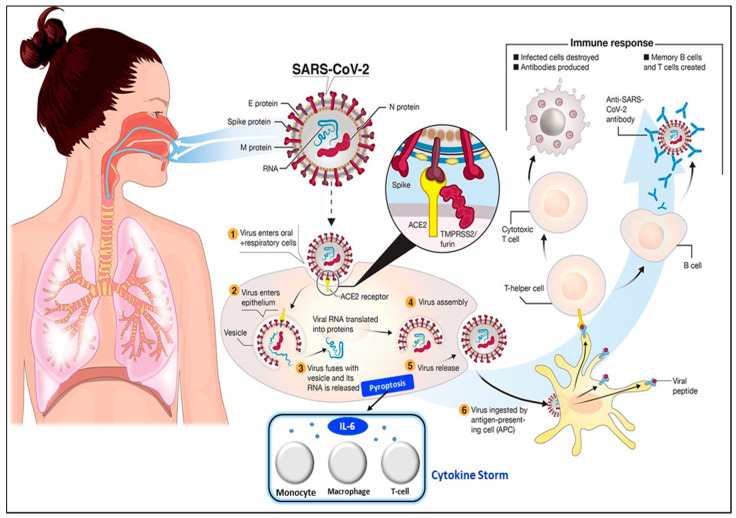
Pathophysiology of the SARS-CoV-2 virus. SARS-CoV-2 virus invasion in the human body undergoes the following processes: (**1**) virus adhesion with ACE2 receptor; (**2**) virus entry into cells; (**3**) release of viral RNA and replication; (**4**) assembly of new viral content, which causes the pyroptosis of the host cell and induces the secretion of pro-inflammatory cytokines and chemokines. These processes attract monocytes, macrophages, and T cells and result in an inflammatory response. (**5**) Release of virus; (**6**) antigen-presenting cells engulf viral particles and activate T helper cells. This activates an adaptive immune response that stimulates B cells to produce antibodies against the virus and T cytotoxic cells to recognize and destroy infected cells. Hence, the accumulation of immune cells at infection sites after an excessive pro-inflammatory stimulus primarily begins with the release of IL-6, which induces the cytokine storm. Reprinted from [27] and modified with the insertion of the cytokine storm part.

The transmission of SARS-CoV-2 into the human body is facilitated by nasal secretions; abiotic surfaces, such as air, water, and atmosphere; feces, and asymptomatic carriers [4]. Upon invading the body, the causative virus binds to angiotensin-converting enzyme-related carboxypeptidase (ACE2) receptors, which are widely present in cardiopulmonary tissues, and then enters the cells [18]. The presence of a pathogen activates the immune system (e.g., innate and adaptive immunity) [19], inducing the release of a large quantity of pro-inflammatory cytokines in a process known as the “cytokine storm” [20]. The cytokine storm starts with the release of IL-6 upon infection with SARS-CoV-2. Then, other cytokines and acute phase proteins, such as vascular endothelial growth factor (VEGF), interleukin-8 (IL-8), interleukin-2R, procalcitonin, and serum ferritin, are released in the blood vessels [21], while SAA and C-reactive protein (CRP) are released in the liver [2,18]. The prolonged inflammatory response of the body leads to acute respiratory distress syndrome (ARD), which results in severe lung injury, decreased level of oxygen saturation, multiple organ failure, and even death [22,23], which are evident in the current pandemic. Hence, the excessive production of pro-inflammatory cytokines in SARS-CoV-2 infection is one of the major factors contributing to the increasing rate of fatality in COVID-19 patients [24,25,26]. The detailed steps of the SARS-CoV-2 virus invasion in the human body are illustrated in Figure 2.

The described pathophysiology is the conventional response of the body’s immune system for pathogenic infection; however, the secretion range of each infection-mediated clinical biomarker in the blood and serum varies between COVID-19 and other infections. This finding is supported by data from clinical studies in which the secretion range of these infection-mediated clinical biomarkers exhibited a specific range for each clinical category of patients. Four distinct clinical categories of patients with SARS-CoV-2 are known—namely mild, moderate, severe, and critical. The severity of the illness and changes in body chemistry can be distinguished in these four categories.

According to the COVID-19 Diagnosis and Treatment Plan issued by the National Health Committee of China [28], patients with mild clinical symptoms and without pneumonia according to radiological imaging are classified as mild patients, whereas patients with fever, respiratory symptoms, and pneumonia according to radiological imaging are categorized as moderate patients. Severe patients are identified with the following criteria: (1) respiratory distress, respiratory rate of ≥30 bpm; (2) peripheral capillary oxygen saturation at rest ≤ 93%; and (3) arterial oxygen partial pressure/fraction of inspiration O_2_ of ≤300 mmHg (1 mmHg = 0.133 kPa). Finally, patients with any one of the following criteria are grouped into the critical category: (1) respiratory failure and mechanical ventilation required, (2) shock, and (3) combination with other organ failures and ICU monitoring and treatment needed [29]. Therefore, the detection and monitoring of biomarkers within a specific concentration range facilitates the diagnosis and increases the understanding of disease severity in an infected individual.

## 3. Infection-Mediated Clinical Biomarkers and Their Electrical Biosensors

This section reviews and highlights potentially effective infection-mediated clinical biomarkers as prognostic and adjunct diagnostic indicators in SARS-CoV-2 infection. The secretion range of each biomarker for four different types of clinical patients (mild, moderate, severe, and critical) was reviewed based on data supported by clinical studies (Table 1) [29,30]. Advancements in electrical biosensors comprising electrochemical and electronics biosensors for these biomarkers are presented in the subsections below. At the end of this section, the applicability of these sensors for COVID-based biosensing applications is assessed and listed as (1) clinically viable, (2) less viable, or (3) not viable in Table 2.

The values shown in Table 1 indicate the mean values and standard deviations of the infection-mediated clinical biomarkers for four different clinical types of patients. The mean values are the indicators for different types of clinical patients. The effective range of concentration for each biomarker is bounded by the upper and lower limits of standard deviation. It is also worth noting that there is an overlap in the standard deviation among different clinical types of patients, especially in IL-6. In this exceptional case, additional supporting data such as symptoms have to be inspected before making conclusions.

### 3.1. Interleukin-6 (IL-6)

#### 3.1.1. Data from Clinical Analysis

IL-6 is activated and produced by a vast range of cells, such as macrophages, monocytes, and dendrites, as a result of the pro-inflammatory response to betacoronavirus infection [18,32]. It is secreted into blood vessels and induces the elevation of IL-6 concentration in the blood. Various studies on the clinical characteristics of COVID-19 patients unveiled an elevation in IL-6 concentration. The concentration of IL-6 is significantly elevated in COVID-19 patients compared with that of healthy individuals, who only exhibited 0–7 pg/mL [33,34]. IL-6 secretion occurs within two hours following onset of infection [35].

By studying the clinical characteristics of the four distinct types of patients, Liu et al. revealed that mild and moderate types of infected patients exhibited IL-6 concentrations of 5.36 ± 1.84 and 13.76 ± 9.07 pg/mL, respectively. Severe and critical patients indicated an elevation in IL-6 to 15.94 ± 14.88 and 33.21 ± 28.58 pg/mL, respectively [29]. Results from a retrospective study revealed that severe and critical types of COVID patients demonstrated IL-6 concentrations of 35.4 and 43.7 pg/mL, respectively [36]. This elevated range of IL-6 in the blood differs between healthy and infected patients. Thus, IL-6 unravels its potential to be implemented as a prognostic biomarker for severity analysis in an infected individual.

A meta-analysis (comprising nine studies with 1426 patients) by Aziz et al. [37] reported a threefold increase in the mean serum concentration of IL-6 (56.8; 41.4–72.3 pg/mL) for patients with severe COVID-19 compared with the 17.3 pg/mL concentration in non-severe patients. Cut-off rates (COF) of more than 55 and 80 pg/mL were disclosed as prognostic indicators of high risk in severe and critical patients, respectively [37,38]. Grifoni et al. [39] demonstrated that IL-6 has the highest sensitivity and specificity as a biomarker for analyzing the severity of COVID-19; the area under the receiver operating curve (ROC) was 0.75 (95% CI 0.64–0.84). Similarly, Meher et al. discovered that IL-6 showed the largest areas under the ROC curve (0.870 and 0.948) for the predictive capability of severity and mortality in COVID-19 patients [32]. Therefore, IL-6 can be used as a prognostic indicator of COVID-19.

#### 3.1.2. Electrical Biosensors for IL-6 Detection

**Figure 3:**
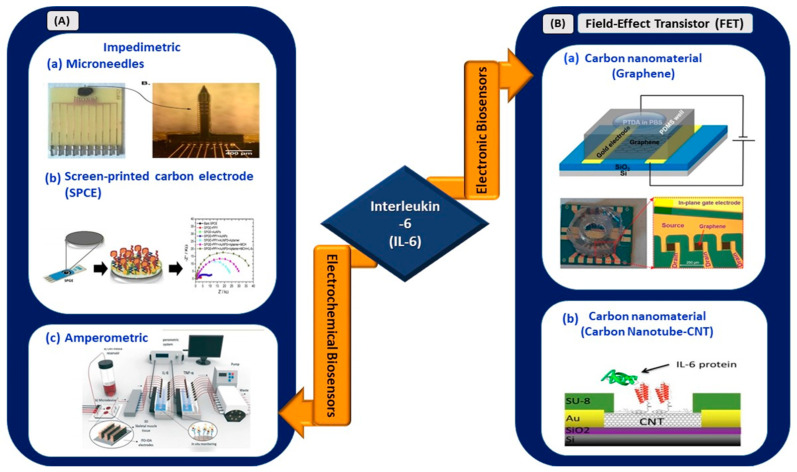
(**A**) Electrochemical biosensors: (**a**) impedimetric detection of IL-6 using microelectrodes with needles structure (reprinted from [40]), (**b**) paper-based impedimetric detection of IL-6 (reprinted with permission from [41]), and (**c**) amperometric sensing system for IL-6 and TNF-α detection (reprinted with permission from [42]). (**B**) Electronic biosensors: (**a**) graphene-based FET biosensor (reprinted from [43]) and (**b**) CNT-based FET biosensor (reprinted from [44]).

In this review, works related to electrical biosensors comprising electrochemical and electronic biosensors are highlighted (Figure 3).

Russell et al. developed an electrochemical sensor made from arrays of microelectrodes on a needle-shaped silicon substrate for the detection of IL-6 in sepsis patients [40]. The sensor exhibited a linear range of 20–100 pg/mL and offered a rapid detection time of 2.5 min, significantly reducing the time compared with the standard laboratory technique, which usually requires 12–72 h. The novel microelectrode structure reduces electrical impedance upon binding with the target because of its needle structure. Thus, it is suitable for in vivo and continuous monitoring of targeted biomarkers and is especially viable for application in the continuous monitoring of IL-6 elevation for COVID-19 patients. Its operating range from 20 to 100 pg/mL is suitable to distinguish severe and critical clinical types of infected patients.

An amperometric sensing system for continuous in situ detection of IL-6 and tumor necrosis factor alpha (TNF-α) was developed by Ortega et al. for studying the progression of various muscular diseases [42]. Cytokines were stimulated under electrical (ITO electrodes) or biological simulation and detected by screen-printed gold electrodes. The flow cell demonstrated its ability to detect IL-6 and TNF-α in the ng/mL range. In addition, Tertis et al. [41] demonstrated the utilization of a nanocomposite made of polypyrrole and gold nanoparticles on a screen-printed carbon electrode (SPCE) for the detection of IL-6 in human sera. Impedimetric measurements revealed the wide linear operating range of the sensor (from 1 pg/mL to 15 µg/mL), with an LOD value of 0.33 pg/mL. The sensor exhibited long-term stability, and 93% of the initial signals were reproducible after 10 days of storage in a dry environment at 4 °C. Therefore, the presence of nanocomposites as the transducing element of an SPCE aids in the sensitive detection of IL-6 compared with unmodified SPCEs. A sensor with a wider operating range, excellent analytical performance, and low LOD at 0.33 pg/mL is feasible for implementation in electrical-based biosensing for IL-6 detection in patients with SARS-CoV-2.

In the context of electronic biosensing, in February 2021, a study by Khan et al. reported a novel approach for the aptamer immobilization of an aptameric GFET biosensor for IL-6 detection [43]. Pyrene-tagged DNA aptamers were implemented for probe immobilization. This method is an alternative to the conventional two-step immobilization technique and eliminates the use of organic solvents, which may cause device malfunction, especially in lab-on-a-chip or POC diagnostic platforms. The sensor exhibited an LOD of 100 pM, which is relatively higher than that obtained through the conventional two-step immobilization of aptamers [45], and further improved, showing an LOD of ~8 pM when the pH of the buffer was changed to 3.64.

In another work, Khosravi et al. used a carbon nanotube microarray-based FET device for the detection of IL-6 in early diagnosed cancer [44]. The sensor demonstrated an LOD of 1, 10, and 10 pg/mL in buffer, blood, and serum, respectively. The detection range from 1 pg/mL to 10 ng/mL revealed the applicability of the sensor for operation in a clinically significant range (2.3 pg/mL to 4 ng/mL) for the diagnosis of early-stage cancer. Hence, this work showed the viability of carbon nanotube microarrays and the PASE functionalization protocol for the development of methods incorporating multiple assays into a single detection platform. In short, this carbon-based sensing platform can be expanded to develop a multiplexed sensing platform for simultaneous detection of various COVID-19 biomarkers.

Therefore, the recent advancements in electrical biosensors for IL-6 detection show an overview of its analytical performance in diagnosing various diseases. The development of IL-6 biosensors for COVID-19 applications requires a definite optimization of its linear operating range and LOD. IL-6 biosensors with an effective operating range from 0 to 61.79 pg/mL (Table 1) are crucial to ensure their viability for application in COVID-19 biosensing platforms.

### 3.2. Vascular Endothelial Growth Factor-D

#### 3.2.1. Data from Clinical Analysis

VEGF is a hypoxia-inducible protein and key regulator of blood vessel formation (angiogenesis) in adult tissues and physiological vascular development during embryogenesis [46]. In mammals, VEGFs are divided into five subdivisions, namely VEGF-A, VEGF-B, VEGF-C, VEGF-D, and placenta growth factor [47]. In the context of COVID-19, VEGF-D plays a significant role because it is a pro-inflammatory dimeric protein that acts as a key factor in acute lung injury and ARD [48]. Individuals with COVID-19 infection show elevated VEGF-D concentrations, which increase vascular permeability and leakiness and aggravate endothelial damage [49,50]. The elevation of VEGF-D concentration has consistently been observed in severe and critical types of infected patients, including ICU and non-ICU patients [25]. A study by Kong et al. revealed the potential use of VEGF-D as a biomarker for evaluating the progression of COVID-19. Results from Python using a random forest machine classifier exhibited the highest forest factor, which was more than 0.14 for VEGF-D compared with 11 clinical indicators and 14 cytokines (TNF-α, SCF, IL-2, IL-4, IL-6, IL-8, IL-10, IL-15, IL-17A, IL-18, IL-1B, and IFN-ɤ) [31]. This result indicates the significance of VEGF-D as an indicator for severity analysis of the disease. The ROC curve for determining the diagnostic accuracy of VEGF-D showed an area under the curve (AUC) higher than 0.836 with a confidence interval (CI) of 95%. Therefore, the elevated levels of VEGF-D in patients with SARS-CoV-2 have the potential to be used as a biomarker for severity analysis in COVID-19 patients.

#### 3.2.2. Electrical Biosensors for VEGF-D Detection

Numerous works that reported VEGF detection were documented by Dehghani et al. in a review article [46]. In January 2021, Lin et al. developed an electrochemical sensor composed of zinc protoporphyrin-modified gold electrodes that self-assembled with avastin and used it for detecting VEGF [51]. The electrodes that underwent light treatment (LT) exhibited a wider detection range of 0.1 pg/mL–10 ng/mL compared with the electrodes that did not (10 pg/mL–10 ng/mL). The sensor exhibited a high sensitivity, detection range, and selectivity for diagnostics and prognostic evaluation in patients with malignancies. However, no biosensor, especially electronic-based biosensor, for sensing VEGF-D has been reported. The development of electrochemical and electronic biosensors for VEGF-D detection has not been explored.

### 3.3. Serum Amyloid A and C-Reactive Protein

#### 3.3.1. Data from Clinical Analysis

CRP and SAA are acute phase proteins secreted in conjunction with inflammatory cytokines in the body upon invasion by infectious pathogens. These proteins have been used as biomarkers for identifying the occurrence of inflammation upon pathogen infection [28]. Compared with CRP, SAA is a highly sensitive infection-mediated biomarker, especially for infections involving lung tissues [30,52,53]. SAA response to virus infection occurs as fast as 24 h, with a rapid 1000-fold increase in plasma concentration [35]. CRP results in a less than 10-fold elevation in plasma concentrations [35]. SAA is induced and released earlier than CRP upon pathogen infection [54].

SAA can be used as an adjunct biomarker for the detection of SARS-related diseases [55,56]. To support this, Zhang et al. [34] proved the viability of SAA as an early biomarker for SARS-CoV-2 detection by evaluating clinical changes in the blood chemistry of 13 infected patients (one mild, eight moderate, three severe, and one critical type of patient). SAA was elevated 20-fold in the initial tests of severe patients compared with a normal range (0–10 mg/L). This substantial elevation in SAA proved the sensitivity and applicability of SAA as an adjunct diagnostic indicator of the infection.

In another study [28], the mean concentrations of SAA and CRP for moderate, critical, and severe patients were studied in 62 patients by Li et al. The results are tabulated in Table 1. Both biomarkers indicated a significant elevation in the plasma concentrations of infected individuals. According to the values reported, SAA concentrations in infectious diseases showed significantly higher increases than CRP concentrations did. This result was further supported by Cheng et al. [30], who showed that SAA exhibited higher sensitivity (96.1%) and specificity (94.3%) than CRP, and 183.6 is an optimal critical value. Thus, with an early and large elevation in plasma, SAA unravels its potential as a sensitive and significant biomarker for the early detection of SARS-CoV-2 infection [57]. Meanwhile CRP can be used an adjunct biomarker for COVID-19 detection.

#### 3.3.2. Electrical Biosensors for SAA and CRP Detection

In recent years, many CRP-related electrical biosensors have been reported for the diagnosis of various diseases and compared with SAA biosensors. In 2015, an optical biosensor utilizing the surface plasmon resonance technique was reported for SAA detection [58]. However, no electrochemical or electronic biosensor for sensing SAA has been reported, and thus, the room for exploring this domain remains open. Therefore, this section focuses on recently reported works on electrical biosensors for CRP detection.

**Figure 4:**
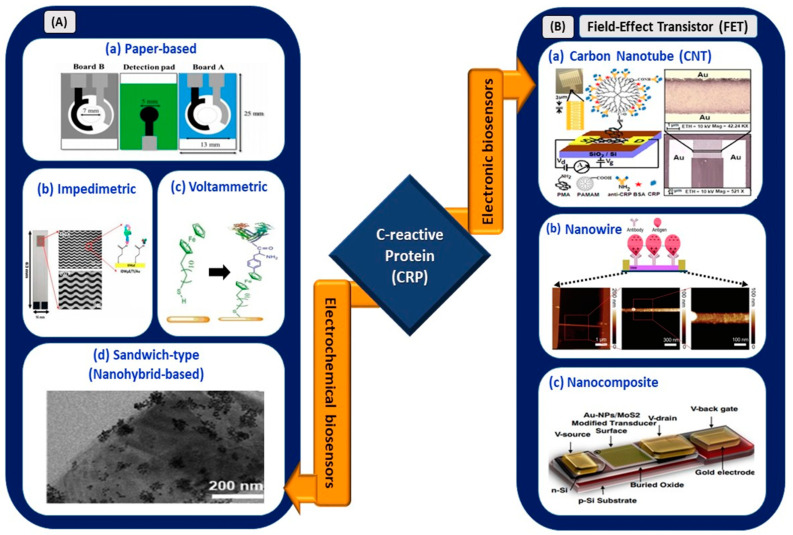
(**A**) Electrochemical biosensors: (**a**) origami paper-based sensing of CRP (reprinted with permission from [59]), (**b**) interdigitated wave-shaped microelectrode-based impedimetric sensor (reprinted from [60]), (**c**) electrochemical sensing of CRP with ferrocene thiol-coated gold electrode (reprinted from [61]), and (**d**) sandwich-type sensor with nanohybrid materials for CRP detection (reproduced from [62]). (**B**) Electronic biosensors: (**a**) PAMAM-modified CNT-based FET (reprinted with permission from [63]), (**b**) nanowire-based FET (reprinted from [64]), and (**c**) nanocomposite-based FET for CRP detection (reprinted with permission from [65]).

Electrochemical sensors utilizing various sensing platforms such as paper-based biosensors; voltammetric immunoassay; impedimetric immunoassay; nanoparticles; quantum dots; sandwich-like biosensors; and FET-based biosensors using carbon-based nanomaterials, nanowires, and nanocomposites are shown in the schematic diagram (Figure 4).

Boonkaew et al. [59] developed a novel origami paper-based electrochemical immunoassay in which a gold nanoparticle (AuNP)-decorated graphene-modified screen-printed carbon electrode (SPCE) was used as a detection medium for CRP in human sera. The sensor exhibited an LOD of 15 ng/mL with a linear range of 0.05–100 µg/mL. However, the incubation time for antibody and antigen conjugation was 50 min, which may not be a favorable condition for rapid sensing of the biomarker in a COVID-based electrical biosensing platform. Similarly, Thangamuthu et al. [66] demonstrated an LOD and linear range of 17 ng/mL and 0.047–200 µg/mL, respectively. However, this paper-based sensor (SPCE with AuNPs) showed an improvement in incubation time, which was only 30 min. This is the first label-free electrochemical sensor reported for the detection of CRP in a drop of serum sample.

An inexpensive and disposable paper-based electrochemical sensor utilizing thiol-terminated poly (2-methacryloyloxyethyl phosphorylcholine) was deployed for CRP detection in serum samples [67]. This sensor offered an improved LOD of 1.6 ng/mL, a wide linear operating range of 5–5000 ng/mL, and an interference-free sensing mechanism. In September 2020, Boonkaew et al. [68] used graphene oxide-modified carbon electrode stencils printed on an ePAD for the simultaneous detection of cardiovascular disease biomarkers, namely CRP, troponin I (cTnI), and procalcitonin (PCT). Although the linear operating range was limited to 1–1000 ng/mL, the LOD showed a significant improvement of up to 0.38 ng/mL.

Dong et al. deposited a composite membrane made of a blended Zr (IV) organic framework with 2,5-thiophenedicarboxylate ligand (Zr-tdc) and ionic liquid (IL) on a carbon paste electrode [69]. This voltammetric sensor exhibited an LOD of 0.2 ng/mL because of the increase in conductivity and surface areas of the adsorption sites. An impedimetric sensor with a 30-micrometer interdigitated wave-shaped microelectrode array (IDWµA) was fabricated by Chinnadayyala et al. [60]. It yielded a broad operation range of 0.1–10,000 ng/mL and LOD values of 0.025 (PBS) and 0.23 ng/mL (human serum). The sensor is free from Warburg diffusion limitation, a condition that may arise from micro-structured fabricated electrodes, and is suitable to be incorporated with a potentiostat render for point-of-care testing. Kuo et al. [70] reported an increase of 85.81% in the sensitivity of electrodes with zig-zag structures at the edges. Therefore, optimization of the design and dimensions of the electrode is a crucial step in determining the electrical performances of devices in biosensing applications.

The graphene quantum dot (QD)-based electrochemical sensor reported by Bing et al. [71] allowed for one-step detection of CRP in blood serum; the sensor had an LOD of 176 pM. In a recent work [61], the efficiency of ferrocene thiol-coated gold for picomolar detection of CRP and BSA was reported. The voltammetric sensor demonstrated an LOD of less than 4 pM for the detection of both biomarkers. In addition, a sandwich-type sensor comprising nanohybrid materials (AuNPs and IL-functionalized molybdenum disulfide (AuNPs/IL-$MoS_{2}$)) exhibited an LOD of as low as 3.3 pg/mL [62]. Therefore, utilization of nanohybrid materials and QDs aids in achieving optimal functioning of the sensing device.

In the context of electronic biosensing, carbon-based nanomaterials, nanowires, and nanocomposites have been used as transducing channel materials of FETs for CRP detection. Rajesh et al. [63] developed a polyamidoamine (PAMAM)-modified single-wall carbon nanotube FET for CRP detection. Immobilization with PAMAM dendrimers offers high sensitivity, as they contribute to the high loading and improvement of probe orientation for coupling with CRP biomolecules. The sensor exhibited an LOD of 85 pM, with a concentration-dependent range of 0.01–10 µg/mL for CRP detection in PBS. In another work, Li et al. [64] implemented a silicon nanowire with a 200-nanometer width and 4-micrometer length as a transducing channel for FET-based cardiac biomarker sensing. This work focused on investigating the settling time involved in biosensing applications, particularly at a low concentration of CRP. The settling time for negatively charged CRP in one solution (pH 8.4) was twice that for positively charged CRP in a solution with pH 7.4. Hence, this parameter has been considered effective in sensing CRP in electronic-based biosensing.

Dalila et al. [65] used a molybdenum disulfide–gold nanoparticle nanocomposite in a back-gated FET for the femtomolar detection of CRP. The presence of the nanocomposite provided large plane properties, which resulted in the stable composition of matter in liquid and gaseous states. Thus, this highly sensitive, selective, and reproducible sensor paves the way for discovering novel methods for early CRP detection. This device is suitable for integration into complementary metal oxide (CMOS) technology for future handheld device applications. Hence, the aforementioned CRP electrical biosensors exhibited an operating range of lower concentrations, with a range from pg/mL up to µg/mL, in detecting various diseases. However, a dynamic operating range from 0.00 to 84.41 mg/mL (Table 1) is required to ensure its applicability in COVID-based electrical biosensing platforms. Thus, the viability of the reported sensors for COVID-19 biosensing applications was assessed and is listed in Table 2.

### 3.4. Serum Ferritin

#### 3.4.1. Data from Clinical Analysis

Serum ferritin is a key mediator of immune dysregulation that results from the cascading effect of pro-inflammatory cytokines [72]. Apart from its conventional role as an iron storage protein, serum ferritin is actively released during infection at the infected site [73]. Previous studies by [74,75,76] signified the importance of serum ferritin as a biomarker in detecting and evaluating the severity of ARD diseases. Qin et al. demonstrated that the level of serum ferritin in patients with severe COVID-19 was fivefold that in healthy individuals, with a range of 452.9–1451.6 ng/mL [77]. In a study by Liu et al. [29], a five- or sixfold increase in serum ferritin level was observed in critical patients compared with mild patients. It was also discovered that serum ferritin increased non-stop, exceeding the upper limit of detection (>2000 µg/mL) in individuals infected with SARS-CoV-2 [78].

Laboratory findings revealed that ferritin significantly elevates serum concentration in patients with mild, moderate, severe, and critical SARS-CoV-2 [29]. The mean values with standard deviations for the respective categories are tabulated in Table 1. Moreover, the clear difference between the secretion and cut-off range acts as a definite indicator for detecting infected patients and determining the severity of the disease [2]. Hence, its distinct concentration elevation in serum due to cytokine storms shows its potential to be implemented as a prognostic and adjunct indicator of COVID-19 [18,79].

#### 3.4.2. Electrical Biosensors for Serum Ferritin Detection

**Figure 5:**
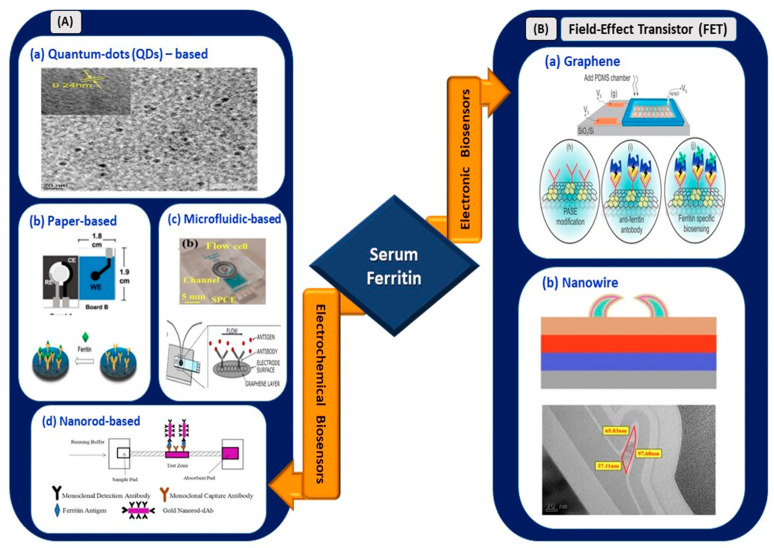
(**A**) Electrochemical biosensors: (**a**) tungsten disulfide quantum dots-based electrochemical detection of serum ferritin (reprinted with permission from [80]), (**b**) handmade paper-based electrochemical sensing of serum ferritin (reprinted with permission from [85]), (**c**) microfluidic-based flow cell for serum ferritin detection (reprinted from [83]), and (**d**) nanorod-based electrical sensing of serum ferritin (reprinted with permission from [84]). (**B**) Electronic biosensors: (**a**) graphene-based FET (reprinted from [87]) and (**b**) nanowire-based FET (reprinted with permission from [86]).

Garg et al. [80] used a myriad of sensing elements for ferritin detection (Figure 5). A biosensor utilizing tungsten disulfide QDs was synthesized and used as an electrochemical sensor for spiked human serum ferritin. The sensor exhibited a linear range of 10–1500 ng/mL and an LOD of 6.048 and 3.800 ng/mL for cyclic voltammetry and direct pulse voltammetry, respectively. Garg et al. [81] used transition metal chalcogenides (TMDs), which were hexagonal boron nitride (HBN) QDs, for sensing serum ferritin. The use of HBN QDs, also known as white graphene, improved the linear range and LOD for serum ferritin detection. A linear range of 10–2000 ng/mL and an LOD of 1.306 ng/mL were retrieved from the sensing application. Hence, the usage of TMD QDs, also known as 2D layered materials, further improved the analytical performance of the device. Their high mechanical strength, thermal stability, and chemical inertness [82] are suitable to be used in electrical biosensing platforms.

Moreover, Garg et al. [83] developed a biosensor utilizing an active screen-printed electrode for continuous flow-based detection of serum ferritin. The sensing system comprised lab-on-chip technology integrated with an amine-functionalized graphene oxide SPCE, which allowed the continuous detection of ferritin in the range of 7.81–500 ng/mL. Although the linear range was lower than the ranges of previously reported techniques, sensing platforms, or materials, a significant improvement in the LOD of the sensor was observed, with a value of 0.413 ng/mL. Song et al. [84] used a gold nanorod reporter probe combined with a natural cotton thread for the sensitive electrochemical detection of human ferritin. The detection exhibited an LOD of 1.58 ng/mL at a response time of 30 min. The linear operating range was from 5 to 5000 ng/mL according to the analytical performance of the sensor. This wider operating range of the sensor is suitable to be implemented in a COVID-19 electrical biosensing platform as it can distinguish the elevated concentration of serum ferritin among different clinical types of patients.

Following that, a novel paper-based analytical device utilizing graphene oxide for serum ferritin detection was fabricated by Boonkaew et al. [85]. The sensor exhibited a linear range of 1–1000 ng/mL and LOD of 0.19 ng/mL. The sensor was stable for up to 3 weeks. This handmade paper-based sensor was implemented for ferritin detection in iron-deficient anemia patients. In the context of electronic biosensing, an FET utilizing polycrystalline-silicon nanowire as a transducing channel material (poly-Si NW FET) exhibited a low LOD limit of 50 pg/mL [86]. The sensor with a 2-micrometer channel gap demonstrated a high sensitivity of 133.47 mV/pH, which exceeded the Nerst limit for the detection of ferritin in buffer solutions and had a wide operating linear range of 50 pg/mL to 500 ng/mL. The first GFET device for detecting ferritin was fabricated by Oshin et al. [87]. The GFET was functionalized with anti-ferritin antibodies for the detection of ferritin in saliva samples. It was found to be a non-invasive detection tool and exhibited an LOD of as low as 10 fM (5.3 ng/mL). In addition, the response rate was within 1–10 s, and the sensor rapidly detected ferritin in the saliva. Thus, it has potential in POC applications. From the electronic devices reported, the FET with poly-Si NW demonstrated an improved LOD and ultra-sensitivity compared to the graphene-based FET. However, the fabrication process involving silicon nanowire is expensive and non-trivial [88,89]. Thus, an FET with a graphene-based biosensing medium is preferable for implementation in the rapid detection of infection-mediated clinical biomarkers.

### 3.5. Procalcitonin

#### 3.5.1. Data from Clinical Analysis

PCT is made of 116 amino acids [90] and is a precursor of calcitonin, which is often triggered and released into the blood vessels through the pro-inflammatory cascading effects of SARS-CoV-2 infection in the human body [91]. PCT is often used as a biomarker for secondary bacterial infection and does not exhibit significant changes during viral infections [92]. However, in the context of COVID-19, Malik et al. demonstrated the viability of PCT to indicate bacterial coinfection and progression toward the severe implication of SARS-CoV-2 infection that leads to pneumonia and ARD [93].

Huang et al. revealed that patients with secondary infections exhibit slight increases in PCT level, which exceeds 0.5 ng/mL. However, the blood biochemistry of most patients was in the normal range—lower than 0.1 ng/mL [25]. Similarly, in [94], PCT concentration increased by 25% in ICU-admitted patients; by contrast, a 0% increase was observed in non-ICU-admitted patients (*P* = 0.029).

In a recent study on PCT levels in 95 patients with SARS-CoV-2 (62 moderate, 21 severe, and 12 critical patients), researchers disclosed fourfold and eightfold increases in PCT levels for severe and critical category patients and moderate category patients, respectively [90]. PCT can be implemented as a biomarker for detecting the severity of SARS-CoV-2 infection in severe and critical category patients compared with mild and moderate types. In addition, serial PCT measurements may be required for accurate prediction of severity compared to single-point measurements [95]. Hence, PCT may be used as a biomarker for the identification of SARS-CoV-2 infection severity, rather than as an adjunct diagnostic biomarker. The reason is its inadequate sensitivity when used in mild and moderate category patients.

#### 3.5.2. Electrical Biosensors for PCT Detection

**Figure 6:**
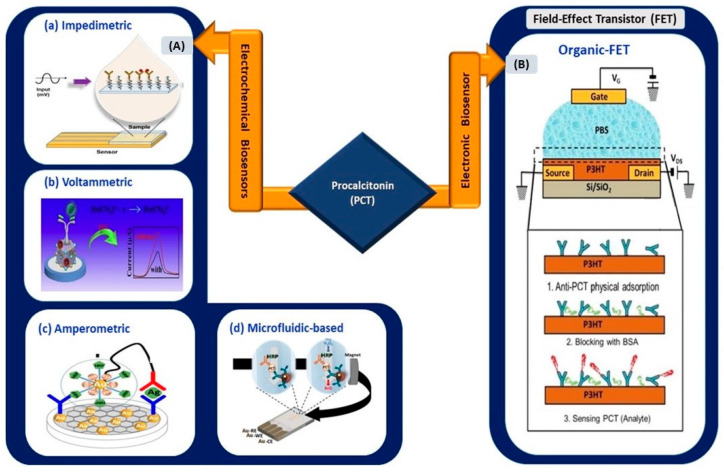
(**A**) Electrochemical biosensors: (**a**) impedimetric sensing of PCT using gold-interdigitated electrodes (reprinted from [98]), (**b**) nanocube-based voltammetric sensor (reprinted with permission from [100]), (**c**) nanocomposite-based amperometric PCT sensor (reprinted with permission from [102]), and (**d**) magneto immunoassay-based microfluidic PCT sensor (reprinted with permission from [99]). (**B**) Organic FET-based electronic biosensor for the detection of PCT (reprinted with permission from [96]).

Shesadri et al. [96] developed an electrolyte-gated organic field-effect transistor (EGOFET), wherein poly-3-hexylthiophene was implemented as the transducing channel material for the immobilization of antibodies and the detection of PCT (Figure 6). As the first electronic-based EGOFET biosensor developed for PCT detection, its fabrication process took only 45 min, and it exhibited an LOD of as low as 2.2 pM. The detection range showed clinical relevance for sepsis diagnosis.

Gherera, A.S. [97] reported the implementation of QDs (zinc sulfide-capped cadmium selenide) on indium-tin-oxide (ITO)-coated glass substrate for PCT detection for urinary tract infection (UTI) tests. The presence of QDs increased the detection range of the sensor from 1 ng/mL to 10 µg/mL, whereas the absence of QDs resulted in a range of 0.1 to 10 µg/mL. The sensing platform was highly stable, retained 90% of its original signal even after 6 weeks of storage, and exhibited an LOD of 0.21 ng/mL. Therefore, the utilization of QDs in a biosensing platform can increase the linear operating range of a sensor. A similar advantage was observed in the work reported by Garg et al. [81]. Tanak et al. [98] used impedimetric sensor-based zinc oxide-modified gold-interdigitated electrodes for PCT detection for sepsis diagnosis. The sensor allowed rapid detection in raw blood samples and human sera, with an LOD of 0.10 µg/mL.

Fernandez et al. [99] conducted a magneto immunoassay, using a microfluidic electrochemical setup. The LOD increased to 0.2 ng/mL, and the detection time was less than 15 min in a 25-microliter sample. Toluidine blue-functionalized NiFe Prussian blue analog nanocubes (NiFe PBA nanocubes@TB) were used as sensing platforms in a voltammetric electrochemical sensor. The LOD was as low as 3 × $10^{-4}$ ng/mL, and the detection range was 0.001–25 ng/mL [100]. Similarly, Abbas et al. [101] used nanostructure-based detection in which cupric tungstate (CUW$O_{4}$) nanospheres with diameters of 200 nm over graphene oxide were used in PCT sensing. The CUW$O_{4}$ and GO formed a superb photoactive heterojunction and improved the LOD to 0.15 pg/mL. In an amperometric-based sensor [102], the use of nanocomposites reduced graphene oxide and AuNPs, and the LOD value was 0.1 pg/mL for PCT detection in human serum. Therefore, with the implementation of nanocomposites, hybrid models increase the ratio of effective surface area to volume for molecular interactions [103] in biosensing applications. Thus, this enhances the electrical performance of the sensing device.

A sandwich-type electrochemical sensor was fabricated by Qu et al. [104]. It demonstrated an ultra-low LOD at femtomolar range (130 fg/mL). Iron sulfide, $Fe_{3}S_{4}$ coupled with Pd nanoparticles deposited on a magnetic glassy carbon electrode, demonstrated excellent electrocatalytic activity and magnetic properties, which increased the sensitivity and stability of the sensor. In a more recent work, a novel sensor using a glassy carbon electrode coated with gold nanoparticles and delaminated sulfur-doped MXene was fabricated for PCT detection [105]. The voltammetric sensor exhibited a lower LOD (0.2 fg/mL), which was lower than that of a previously reported work. However, the linear range of the sensor was limited to 0.01–1.0 pg/mL. Qu et al. [104] presented a wider linear detection window ranging from 500 fg/mL to 50 ng/mL. The sensor detected PCT in both human serum and buffer solution and exhibited an LOD of 130 fg/mL. Therefore, by referring to the elevated concentration of PCT in Table 1, electrical biosensors with an operation range up to 50 ng/mL are suitable to be applied in COVID-based electrical biosensing platforms.

In order to summarize the biosensors reviewed above, the performance aspects and their viability for COVID-based electrical biosensing applications are presented in Table 2. The viability of each biosensor for COVID-19 biosensing applications was assessed based on their linear operating range to determine their suitability to detect the targeted biomarkers in the range as listed in Table 1. The viability of each biosensor is denoted as either (1) clinically viable, (2) less viable, or (3) not viable in the table below.

**Table 2:** Performance aspects and viability assessment of recently reported electrical biosensors associated with infection-mediated clinical biomarkers.

Infection-Mediated Clinical Biomarkers	Electrical Biosensors (Types)		Sensing Material	Limit of Detection (LOD)	Linear Operating Range	Samples	Viability Assessment (COVID-19)	Ref
Interleukin-6 (IL-6)	Electrochemical	Impedimetric	Gold SPE	20 pg/mL	20–100 pg/mL	Buffer, human serum	Less viable	[40]
			Nanocomposites	0.33 pg/mL	1 pg/mL– 15 µg/mL	Human serum	Clinically viable	[41]
		Amperometric	Gold SPE	8 ng/mL	N/A	3D tissues	Not viable	[42]
	Electronic	Field-effect transistor (FET)	Graphene	8 pM	N/A	Buffer	Clinically viable	[43]
			Carbon nanotube (CNT)	1 pg/mL (buffer), 10 pg/mL (blood), 10 pg/mL (serum)	1 pg/mL–10 ng/mL	Buffer, blood, serum	Clinically viable	[44]
VEGF-D	Electrochemical	Amperometric	Zinc protoporphyrin-modified gold electrodes	N/A	0.1 pg/mL–10 ng/mL	Human protein	Not viable	[51]
C-reactive Protein (CRP)	Electrochemical	Paper-based	Graphene-modified SPCE	15 ng/mL	0.05–100 μg/mL	Human serum	Not viable	[59]
			Gold particle-modified SPCE	1.6 ng/mL	5–5000 ng/mL	Serum	Not viable	[67]
			Gold nanoparticle-modified SPCE	17 ng/mL	0.047–3.6 µg/ mL	Human serum and blood	Not viable	[66]
			Graphene oxide (GO)	0.38 ng/mL	1–1000 ng/mL	Serum	Not viable	[68]
		Sandwich-type	Nanohybrid materials (Au NPs/IL-MoS2)	3.3 pg/mL	0.01–100 ng/mL	Serum	Not viable	[62]
			Silica microspheres	0.0017 ng/mL	0.005–125 ng/mL	Human serum	Not viable	[106]
		Voltammetric	Composite membrane on carbon electrode	0.2 ng/mL	N/A	Serum	Not viable	[69]
			Ferrocene- terminated monolayer on a gold electrode	4 pM	1–10,000 pM	Buffer	Not viable	[61]
		Impedimetric	Self-assembled monolayer (SAM)	0.025 ng/mL (buffer), 0.23 ng/mL (human serum)	0.01–10,000 ng/mL	Human serum and buffer	Not viable	[60]
		Quantum dots (QDs)	Graphene QDs	176 pM	0.5–70 nM	Blood serum	Not viable	[71]
	Electronic	Field-effect transistor (FET)	Carbon nanotubes (CNTs)	85 pM	0.01–10 µg/mL	PBS	Not viable	[63]
			Silicon nanowire	N/A	100 ng/mL–10 µg/mL	N/A	Not viable	[64]
			Nanocomposite (Au-NPs/MoS2)	8.38 fg/mL	10 ng/mL–100 pg/mL	Buffer	Not viable	[65]
Serum Ferritin	Electrochemical	Quantum dots (QDs)	Tungsten disulfide (WS2-B)	DPV: 3.800 ng/mL CV: 6.048 ng/mL	10–1500 ng /mL	Human serum	Clinically viable	[80]
			hBN (hexagonal boron nitride)	1.306 ng/mL	10–2000 ng/mL	N/A	Clinically viable	[81]
		Microfluidic	Amine-functionalized graphene oxide on SPCE	0.413 ng/mL	7.81–500 ng/mL	Human serum	Less viable	[83]
		Paper-based	Graphene oxide	0.19 ng/mL	1–1000 ng/mL	Human serum	Clinically viable	[85]
		Nanorod-based	Gold nanorod- reporter probe	1.58 ng/mL	5–5000 ng/mL	Human ferritin	Clinically viable	[84]
	Electronic	Field-effect transistor (FET)	Graphene	10 fM 5.3 ng/L	5.3 ng/L–0.5 µg/L	Saliva	Clinically viable	[87]
			Silicon nanowire	50 pg/mL	50 pg/mL–500 ng/mL	Buffer	Less viable	[86]
Procalcitonin (PCT)	Electrochemical	Impedimetric	Zinc oxide-modified gold-interdigitated electrodes	0.10 µg/mL	0.01–10 µg/mL	Human serum and blood	Not viable	[98]
		Amperometric	Nanocomposites (GO and AuNPs)	0.1 pg/mL	0.05–100 ng/mL	Human serum	Clinically viable	[102]
		Quantum dots (QDs)	Zinc sulfide-capped cadmium selenide on ITO	0.21 ng/mL	0.1 ng/mL–10 µg/mL (with QDs)	Synthetic PCT, human serum	Clinically viable	[97]
		Microfluidic-based	Magneto immunoassay	0.2 ng/mL	0.05 –100 ng/mL	Serum, plasma, buffer	Clinically viable	[99]
		Voltammetric	NiFe PBA nanocubes	3 × 10^–4^ ng/mL	0.001–25 ng/mL	Serum	Clinically viable	[100]
			d-S-Ti3C2TX MXene-doped glassy carbon electrode	0.2 fg/mL	0.01–1.0 pg/mL	Buffer	Not viable	[105]
		Sandwich-type	Pd NP on magnetic glassy carbon electrode	130 fg/mL	500 fg/mL– 50 ng/mL	Human serum, PBS buffer	Clinically viable	[104]
	Electronic	EGOFET (electrolyte-gated organic field-effect transistor)	Poly-3-hexylthiophene (P3HT) organic semiconductor surface	2.2 pM	0.8 pM–4.7 nM	PBS buffer	Clinically viable	[96]

SPCE: Screen-printed carbon electrode. SPE: Screen-printed electrode. N/A: Not available.

## 4. Insights into Infection-Mediated Clinical Biomarkers and Their Electrical Biosensors in COVID-19 Electrical Biosensing Platforms

Inflammatory cytokines and acute phase proteins released because of a “cytokine storm” can be used as prognostic and adjunct diagnostic indicators of COVID-19. The relative changes in SAA and CRP concentrations in the four clinical types of patients exhibited higher ranges than those in infection-mediated clinical biomarkers. However, SAA is an efficient adjunct biomarker for COVID-19 diagnosis as it is secreted earlier than CRP is. The 1000-fold elevation of SAA concentration in the blood within 24 h of infection sets a benchmark for the sensitive detection of SARS-CoV-2 in the body [35].

Serum ferritin, PCT, and VEGF-D exhibited relative changes in concentrations from 0.012 to 5.700. These distinctive differences in concentration between healthy and infected individuals unveil the possibility of implementing infection-mediated biomarkers as adjunct diagnostic indicators for SARS-CoV-2 infection. These biomarkers can be used as indicators in asymptomatic or pre-symptomatic individuals from epidemic locations. They can also be used as adjunct indicators for detection in patients with symptoms who obtained false negative results in nucleic acid- and antigen–antibody-based detection techniques. Each biomarker exhibited a definite range of concentrations for the four different clinical categories of patients, as shown in Table 1. Thus, these distinctive ranges of concentrations demonstrated their viability to evaluate the severity of the disease, especially IL-6, PCT, and VEGF-D, which showed more significant roles as severity indicators than as diagnostic indicators of COVID-19 infection. It is worth noting that the concentrations of IL-6 for healthy (0.00–7.00 pg/mL) and mild (5.36 ± 1.84 pg/mL) types of COVID patients overlap with each other. Therefore, in this exceptional case, additional factors such as symptoms and results from other diagnostic techniques have to be inspected before concluding. Hence, infection-mediated clinical biomarkers can be implemented as prognostic and adjunct diagnostic indicators in COVID-19 infection.

On the other hand, electrical biosensors, especially electronic-based biosensing platforms, exhibited more advantages compared to electrochemical sensors. Electronic-based biosensors offer ultra-sensitive, rapid, low-cost, and handheld technology possibilities for the detection of these infection-mediated clinical biomarkers. In contrast, electrochemical biosensors demand complex, expensive, and time-consuming procedures for assay preparations. Thus, this marks the suitability of electronic biosensors for developing COVID-19 biosensors as they require a fast and sensitive medium for diagnostic and prognostic analyses of the causative virus infection. The use of 2D nanomaterials, nanocomposites, hybrid materials, and QDs increases the optimal functioning of the biosensing device by improving its electrical characteristics. The linear operating range and LOD of biosensors are further improved with the implementation of these materials as the transducing materials. However, the dynamic operating range of a biosensor is an important parameter to ensure its viability to be implemented in COVID-19 biosensing applications. Biosensors targeting a wide linear operating range from pg/mL to mg/mL are required for sensing the aforementioned infection-mediated clinical biomarkers in COVID-19 applications.

Therefore, FET-based electronic biosensors could be implemented in COVID-based electrical biosensing platforms as they offer direct translation for the interaction between targeted molecules and probes and a rapid detection time as low as 1–10 s [87]. The incorporation of 2D nanomaterials, especially carbon-based nanomaterials, as the transducing channel material elevates the sensitivity of a sensing platform to its surrounding charges. Hence, the use of the GFET device developed by our group for ultra-sensitive detection is proposed for the detection of these infection-mediated clinical biomarkers [107,108]. This optimized single-layer graphene-based sensing platform exhibited an LOD of as low as ag/mL ($10^{-18}$) and a linear detection range from pg/mL to mg/mL for the effective sensing of anti-diuretic hormone. Hence, this ultra-sensitive GFET platform can be used for the detection of infection-mediated clinical biomarkers with concentrations from pg/mL to mg/mL ranges in blood and plasma samples.

**Figure 7:**
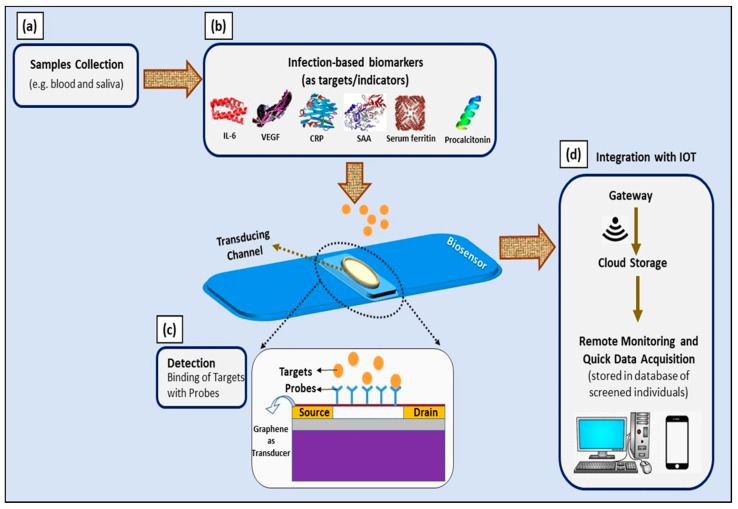
Schematic illustration of utilizing infection-mediated clinical biomarkers as adjunct diagnostic and prognostic indicators in developing electrical-based COVID-19 biosensors for point-of-care application. (**a**) Samples collected from infected individuals; (**b**) infection-mediated clinical biomarkers comprising IL-6, VEGF-D, CRP, SAA, serum ferritin, and procalcitonin as targets; (**c**) graphene-based FET device as a biosensor and electrical changes upon binding of targets with probes, measured with three-point probe measurements; and (**d**) integration with IoT for remote monitoring and quick data acquisition.

Moreover, the aforementioned ultra-sensitive GFET device can be implemented as a multiplex sensing platform to simultaneously detect all of these biomarkers (IL-6, VEGF-D, CRP, SAA, serum ferritin, and PCT). Its wide linear operating range is suitable for the simultaneous detection of all of these biomarkers; thus, multiplex detection is essential to ensure the accuracy of diagnosis and prognosis of COVID-19 infection. The suggested platform could be extended by the incorporation of Internet of Things—particularly smartphones and specific smartphone applications that allow remote monitoring and on-site data analysis by medical and government authorities [109]. The schematic representation of this idea is presented in Figure 7.

## 5. Conclusions

The deleterious COVID-19 pandemic has affected many households and led to a global crisis. Data from various clinical studies and meta-analyses confirmed the applicability of the infection-mediated clinical biomarkers IL-6, VEGF-D, SAA, CRP, serum ferritin, and PCT as prognostic and adjunct diagnostic indicators for developing electrical-based COVID-19 biosensors. The distinctive secretion ranges of the infection-mediated clinical biomarkers for four different types of clinical patients exhibited potential in evaluating the severity of the illness and the progression of the infection. The studies on electrical biosensors associated with these biomarkers showed the advantages of sensitive, selective, and reliable biomarkers. Implementing electrical-based biosensors, especially FETs, allows label-free, direct detection and minimal usage of samples and reduces unwanted transmission of viral content during the detection process. In conclusion, infection-mediated clinical biomarkers with these advantages are effective prognostic and adjunct diagnostic indicators of COVID-19, and utilizing electrical biosensors as detection platforms is one efficient approach for managing the outbreak.

## Figures and Tables

**Table 1:** Concentrations of infection-mediated clinical biomarkers in normal and four clinical types (mild, moderate, severe, and critical) of COVID-19 patients.

Infection-Mediated Clinical Biomarkers	Interleukin-6 (IL-6) ^a^ (pg/mL)	Vascular Endothelial Growth Factor (VEGF) ^b^ (mg/mL)	Serum Amyloid-A (SAA) ^c^ (mg/mL)	Serum Ferritin ^d^ (ng/mL)	C-Reactive Protein (CRP) ^e^ (mg/mL)	Procalcitonin (PCT) ^f^ (ng/mL)
Baseline (normal range in a healthy individual)	0.00–7.00	27.00–30.00	0.00–10.00	15.00–150.00	0.00–1.00	0.00–0.10
**Clinical Categories (mean + standard deviation)**						
Mild	5.36 ± 1.84	N/A	N/A	148.22 ± 196.53	7.79 ± 10.88	N/A
Moderate	13.76 ± 9.07	N/A	123.57 ± 75.81	396.02 ± 260.29	12.79 ± 7.70	0.08 ± 0.279
Severe	15.94 ± 14.88	25.90 (12.30, 40.60)	171.91 ± 56.89	469.53 ± 188.18	17.84 ± 15.09	0.14 ± 0.353
Critical	33.21 ± 28.58	62.90 (45.80, 79.60)	181.00 ± 40.66	1,005.16 ± 577.32	46.75 ± 37.66	0.44 ± 0.512

^a,d,e^ Data reported by Liu et al. [29] (*n* = 65 patients). ^b^ Data reported by Kong et al. [31] (*n* = 24 patients). ^c,f^ Data reported by Li et al. [28] (n = 132 patients).

## Data Availability

Not applicable.
